# A weighted *q*-gram method for glycan structure classification

**DOI:** 10.1186/1471-2105-11-S1-S33

**Published:** 2010-01-18

**Authors:** Limin Li, Wai-Ki Ching, Takako Yamaguchi, Kiyoko F Aoki-Kinoshita

**Affiliations:** 1 Advanced Modeling and Applied Computing Laboratory, Department of Mathematics, The University of Hong Kong, Pokfulam Road, Hong Kong; 2 Department of Bioinformatics, Faculty of Engineering, Soka University, Tokyo, Japan

## Abstract

**Background:**

Glycobiology pertains to the study of carbohydrate sugar chains, or glycans, in a particular cell or organism. Many computational approaches have been proposed for analyzing these complex glycan structures, which are chains of monosaccharides. The monosaccharides are linked to one another by glycosidic bonds, which can take on a variety of comformations, thus forming branches and resulting in complex tree structures. The *q*-gram method is one of these recent methods used to understand glycan function based on the classification of their tree structures. This *q*-gram method assumes that for a certain *q*, different *q*-grams share no similarity among themselves. That is, that if two structures have completely different components, then they are completely different. However, from a biological standpoint, this is not the case. In this paper, we propose a weighted *q*-gram method to measure the similarity among glycans by incorporating the similarity of the geometric structures, monosaccharides and glycosidic bonds among *q*-grams. In contrast to the traditional *q*-gram method, our weighted *q*-gram method admits similarity among *q*-grams for a certain *q*. Thus our new kernels for glycan structure were developed and then applied in SVMs to classify glycans.

**Results:**

Two glycan datasets were used to compare the weighted *q*-gram method and the original *q*-gram method. The results show that the incorporation of *q*-gram similarity improves the classification performance for all of the important glycan classes tested.

**Conclusion:**

The results in this paper indicate that similarity among *q*-grams obtained from geometric structure, monosaccharides and glycosidic linkage contributes to the glycan function classification. This is a big step towards the understanding of glycan function based on their complex structures.

## Background

Glycobiology pertains to the study of carbohydrate sugar chains, or glycans, in a particular cell or an organism. Glycans consist of monosaccharides, which are linked to other monosaccharides by a glycosidic bond, which can take on a variety of conformations. Thus a single monosaccharide may be linked to at most four other monosaccharide (children), in addition to its parent monosaccharide. Glycans are usually found on proteins and lipids, linked from its root monosaccharide and branching out. The structure at the leaves are understood to be important for various biological functions [1]. These often consist of many branches and can become rather complex. Although major glycan motifs (often found at the leaves) are known to be biomarkers for specific diseases, the mechanism of this glycan recognition is still not well understood. Bioinformatics methods for glycobiology have recently developed rapidly due to the availability of glycan structure databases provided by major institutions including KEGG and the CFG (Consortium for Functional Glycomics). One of the important bioinformatics techniques applied to glycans is support vector machines (SVMs) for the extraction of species-specific glycan substructures [2]. This method has been further applied to the extraction of leukemia-specific glycan motifs in human [3]. The application of tree kernels for glycan classification [4] was then developed at the same time as the *q*-gram distribution kernel for disease-specific glycan motif extraction [5].

These methods were all groundbreaking in the sense that glycan structures have complex tree structures and that no one had ever tried, let alone were successful, in finding biologically important glycan motifs from glycan structure data alone. However, glycobiology is still a difficult topic, since these methods have not been further improved nor applied to actual problems in glycobiology. One of the reasons for this lay in the fact that, just as amino acids have physico-chemical properties that allow them to be grouped with one another, the components of glycans (monosaccharides and glycosidic linkages) also have such properties which have been ignored in the previous methods.

Thus, in this paper, we attempt to incorporate such similarity measures in glycan kernels in order to increase the classification accuracy of glycan structures, and thus improve glycan function prediction. Namely, we focused on the *q*-gram kernel due to the large variety of features that can be handled with a fairly simple kernel. We incorporated similarity measures as resolved by the KCaM (KEGG Carbohydrate Matcher) algorithm [6], a dynamic programming method for tree structure alignment. We then compared the accuracy of our new method compared to the previous methods and show that the prediction can be improved by using our proposed weighted *q*-gram method.

## Methods

We apply the Support Vector Machine (SVM) to classify glycans based on different kernels constructed from their tree structures. We first describe the original *q*-gram method and then our proposed weighted *q*-gram method. We constructed three kernels for the weighted *q*-gram method: Linkage (LK) kernel, KCaM (KM) kernel and Linkage KCaM (LKM) kernel. Linkage kernel considers the similarity of glycosidic linkage, their layers and the tree structure among *q*-grams. In previous work, the concept of *layers *was used to indicate the distance of a particular monosaccharide from the root. We also make use of this concept in our kernels by defining the layer of a monosaccharide and its linkage towards its parent as the number of linkages between the monosaccharide and the root. KCaM kernel considers the similarity of tree structure among different *q*-grams. Linkage KCaM kernel further improves KCaM kernel by incorporating glycosidic linkage and layer similarity of *q*-grams.

### The *q*-gram method

Suppose we are given *n *glycans *G *= {*g*_1_,⋯, *g*_*n*_}. A *q*-gram is defined as a subtree with *q *nodes isomorphic to a path, in which every node has no more than two adjacent nodes, for *q *≥ 1. We denote the set of all *q*-grams existing in these *n *glycans to be *q*-gram set  where *d*_*q *_is the total number of *q*-grams in the set Φ_*q*_. Let *Q*-gram set Φ = {Φ_*q*_}_*g*∈*Q*_, where *Q *is a set of integers, and we usually take *Q *= {2,⋯,9}. A *q*-gram is also called as *Q*-gram if *q *∈ *Q*. Then *Q*-gram set Φ is the set of all the *Q*-grams existing in the *n *glycans. For each glycan *g*_*s*_, the *q*-gram representation is a column vector , where  means the number of *i*th *q*-gram  in the glycan *g*_*s*_, while the *Q*-gram representation is the column vector **x**_**s**_, which is the concatenation of the vectors , where *q *∈ *Q*. Let  and *X *= [**x**_**1**_,⋯,**x**_**n**_] ∈ *R*^*d*×*n*^, where *d *is the total number of *Q*-grams in the set Φ. We note that *d *= ∑_*q*∈*Q*_*d*_*q*_. The feature space Γ_*q*_using *q*-grams is spanned by the column vectors of *X*_*q*_, and the feature space Γ_*Q *_using *Q*-grams is spanned by the column vectors of *X*. The feature space Γ_*Q *_is the direct sum of feature spaces Γ_*q *_for all *q *∈ *Q*, i.e., Γ_*Q *_= ⊕_*q*∈*Q *_Γ_*q*_. The *q*-gram similarity between glycan *g*_*s *_and *g*_*t *_can be represented as the inner product of their feature vectors,

Thus the *q*-gram kernel *K*_*q *_can be simply obtained by  The *Q*-gram kernel *K*_*Q *_can then be obtained by *K*_*Q *_= *X*^*T*^*X*. We note that it is easy to see that *K*_*Q *_= ∑_*q*∈*Q*_*K*_*q*_.

### Weighted *q*-gram method

We note that the *q*-gram method does not consider the similarity among the *q*-grams in Φ_*q*_. Suppose the similarity among the *q *-grams in Φ_*q *_is represented as a weight matrix *W*_*q*_, where  is the similarity score between the *i*th *q*-gram and *j*th *q*-gram. We represent the similarity between the two glycan *g*_*s *_and *g*_*t *_as follows:

Thus the weighted *q*-gram kernel can be obtained by . The weighted *q*-gram method is indeed a generalization of *q*-gram method. The *q*-grams and *p*-grams are considered to have no similarity if *p *is not equal to *q*. One can then obtain the weighted *Q*-gram kernel by

If we add a weight *α*_*q *_on each *q*-gram, we can get double weighted *Q*-gram kernel *K*_*αwQ *_= ∑_*q*∈*Q*_*α*_*q*_*K*_*wq*_. Here *W*_*q *_is required to be a positive definite matrix since the kernel matrix should be positive definite. Let . The feature space Γ_*wq *_for weighted *q*-gram method is spanned by the column vectors of *S*_*q*_*X*_*q*_, and the feature space for the weighted *Q*-gram method is Γ_*wQ *_= ⊕_*q*∈*Q *_Γ_*wq*_.

### *q*-gram similarity

In this section, we discuss some methods to obtain the similarity among the *q*-grams.

#### Linkage kernel

Each *q*-gram is composed of several monosaccharides and bonds. We represent the *i*th *q*-gram by , where *l*^*i *^is the layer of this *q*-gram, *σ*^*i *^represents the structure shape of this *q*-gram,  is an ordered set of monosaccharides,  is an ordered set of bonds.

For each *q*, the *q*-grams with different structure shape is considered to be totally different.

Now we consider two *q*-grams,  and . We denote the similarity between them by *S*_*q*_(*i*, *j*). As mentioned above, if *σ*^*i *^and *σ*^*j *^is not same, then *S*_*q*_(*i*, *j*) = 0. The similarity between the *q*-grams with the same structure shape depends on the similarity of the layers, monosaccharides, and bonds between them. We represent the similarity between *q*-grams  and  by

where *S*^*l*^(*l*^*i*^, *l*^*j*^) is the similarity of the layers of the two *q*-grams,  is the similarity of the monosaccharides  and , and  is the similarity of the bonds  and .

For the similarity between the layers, it is easy to obtain the similarity function by using the distance of the layers. We then define the similarity

After the linkage similarity *S*_*q *_is constructed for all *q*-grams, the linkage kernel can be obtained similarly as discussed in the previous section. This method of measuring the similarity among *q*-grams is denoted as the LK method. For the monosaccharides and bonds, since there are too many possibilities of combinations, in the database, we only choose the most frequent ones, which appear most in mammals. In the KEGG glycan database, the most frequent monosaccharides (including other chemical compounds) and the bonds are shown in Table 1. The similarity among monosaccharides can be obtained from the chemical structure comparison method SIMCOMP developed by Hattori et al. [7,8]. The bonds similarity is set by their chemical meanings and is shown in Table 2.

**Table 1:** Statistics about the chemical bonds and monosaccharides in glycan database.

bond	occurrence	percent	mono	occurrence	percent
b1-4	16475	0.272	GlcNAc	13110	0.1833
a1-3	7002	0.1156	Gal	12248	0.1712
b1-3	6039	0.0997	Man	10632	0.1486
a1-6	4802	0.0793	Glc	7446	0.1041
b1-2	4051	0.0669	LFuc	3003	0.042
a1-2	3974	0.0656	Neu5Ac	2682	0.0375
a1-4	3734	0.0617	S	2653	0.0371
b1-6	2538	0.0419	GalNAc	2601	0.0364
a2-3	1692	0.0279	LRha	1606	0.0225
-6	1249	0.0206	Xyl	1418	0.0198
a2-6	1217	0.0201	GlcA	1135	0.0159
-2	1042	0.0172	GlcN	1074	0.015
b1-	879	0.0145	*	999	0.014
b1-1	809	0.0134	Cer	833	0.0116
a1-	600	0.0099	P	772	0.0108
-4	585	0.0097	Lgro-manHep	589	0.0082
-3	553	0.0091	Asn	545	0.0076
-	318	0.0053	Kdo	496	0.0069
a1-5	315	0.0052	Fruf	358	0.005
a2-8	224	0.0037	LIdoA	354	0.0049
1-3	223	0.0037	GalA	337	0.0047
1-	220	0.0036	LAraf	309	0.0043
1-4	218	0.0036	Neu5Gc	253	0.0035
a2-4	152	0.0025	Galf	237	0.0033

Occurrence gives the times each chemical bond and monosaccharides appears in glycan database. Percent means the percentage of each chemical bond or monosaccharides in glycan database.

**Table 2:** Bond similarity. The matrix gives the similarity among chemical bonds in glycan database. Higher score indicates that the two chemical bonds are more similar to each other.

	a1-2	a1-3	a1-4	a1-6	b1-2	b1-3	b1-4	b1-6	a2-3	a2-6	a2-8	a2-9	-6	-3	-4
a1-2	1														
a1-3	0.6	1													
a1-4	0.6	0.9	1												
a1-6	0.6	0.6	0.6	1											
b1-2	0.8	0.5	0.5	0.5	1										
b1-3	0.5	0.8	0.6	0.5	0.6	1									
b1-4	0.5	0.6	0.8	0.5	0.6	0.9	1								
b1-6	0.5	0.5	0.5	0.8	0.6	0.6	0.6	1							
a2-3	0.4	0.4	0.4	0.4	0.4	0.4	0.4	0.4	1						
a2-6	0.4	0.4	0.4	0.4	0.4	0.4	0.4	0.4	0.6	1					
a2-8	0.4	0.4	0.4	0.4	0.4	0.4	0.4	0.4	0.6	0.7	1				
a2-9	0.4	0.4	0.4	0.4	0.4	0.4	0.4	0.4	0.6	0.7	0.85	1			
-6	0	0	0	0.55	0	0	0	0.3	0	0	0	0	1		
-3	0	0.47	0	0	0	0.41	0	0	0	0	0	0	0	1	
-4	0	0	0.8	0	0	0	0.2	0	0	0	0	0	0	0	1

#### KCaM kernel and Linkage KCaM kernel

KCaM is a tool implementing a polynomial-time dynamic programming algorithm to align glycan tree structures. From KCaM we can obtain the similarity score for two glycan structures in the range of 0 to 100. Here we apply KCaM to the *q*-gram structures to obtain the similarity scores among the *q*-grams. We denote this method of measuring *q*-gram similarity as the KM method.

We note that KCaM also does not consider the similarity of the monosaccharides and bonds among the glycan structures and the layers of *q*-grams. However, a method of computing the score matrices of monosaccharides and linkages have been developed, which can be utilized by KCaM [9]. Thus we incorporated the linkage similarity and layers into the score matrix and developed a new KCaM method called Linkage KCaM (LKM) method. We then use the alignment scores of all *q*-grams obtained by the Linkage KCaM as the their similarity scores.

Let the alignment score matrix obtained by KCaM or Linkage KCaM be *S*_*q*_. Here *S*_*q*_(*i*, *j*) is the alignment score between the *i*th and *j*th *q*-grams. Then the weight matrix *W*_*q *_can be represented using *S*_*q*_. Since the kernel  should be positive definite, we revise *W*_*q *_to  Thus we have the kernel 

## Results and discussion

### Glycan data

We used two sets of glycan data to evaluate the classification performance using different kernels we constructed. Glycan structure data was retrieved from the KEGG/GLYCAN database [10] and their annotations are from CarbBank/CCSD database [11]. The first data set includes glycan structures that are known to be related to leukemic cells, as was originally analyzed by [3]. We also tested our method on another data set of glycans related to cystic fibrosis as was previously used by [5]. The data used here is summarized in Table 3. The statistical analysis for all *q*-grams in the glycan database shows the distribution of each monosaccharide and chemical bond in Table 1. The most frequent chemical bond in the glycan database is *b*1 - 4, and the most frequent monosaccharide is GlcNAc. The similarity among monosaccharides is calculated by the software SIMCOMP [7,8], and the similarity among chemical bond is obtained by their chemical meanings, which is shown in Table 2.

**Table 3:** Glycan data. The data labels, the number of each class and the total number of each data.

leukemia	non-leukemia	total
162	193	355

cystic fibrosis	non-cystic	total

104	118	222

## Results

We evaluated the performance of our weighted *q*-gram method by comparing it with the original *q*-gram method on glycan data. SVM was performed in the environment of Matlab version 2007a. Table 4 shows the Area Under the ROC curve(AUC) using SVM classifier for the two datasets. The AUC values in Table 4 are the average of 5-fold cross validation performed 50 times. We used two ways to combine the information from different *q*-gram representations. First is the *Q*-gram method, which represents each glycan by the vector of {2,⋯,9}-grams. The other is the recently developed multiple kernel learning [12], which generates an optimal weighting of each representation for each classification task. In Table 4, for the traditional *q*-gram method, KCaM kernel method and Linkage KCaM kernel method, the results using *Q*-gram and multiple kernel method are both reported. For the linkage kernel method, we only reported the results for Linkage 2-gram and Linkage 3-gram since when *q *is larger than 3, the *q*-gram similarity calculation becomes exponential.

**Table 4:** Results. The results for four methods are reported: traditional *q*-gram, Linkage (LK) kernel method, KCaM (KM) kernel method and Linkage KCaM (LKM) kernel method.

Leu	*q*-gram	KM	LKM	LK	Cystic	*q*-gram	KM	LKM	LK
2-gram	0.9578	0.9555	0.9623	0.9606	2-gram	0.7872	0.7666	0.7581	0.7684
3-gram	0.9568	0.9608	0.9621	0.9647	3-gram	0.8220	0.8151	0.8034	0.7823
4-gram	0.9499	0.9516	0.9540		4-gram	0.7812	0.7648	0.7467	
5-gram	0.9354	0.9365	0.9311		5-gram	0.7254	0.7530	0.7441	
6-gram	0.9300	0.9272	0.9287		6-gram	0.6886	0.7224	0.7265	
7-gram	0.9272	0.9245	0.9181		7-gram	0.5965	0.6088	0.6186	
8-gram	0.9086	0.9039	0.8990		8-gram	0.5319	0.5354	0.5522	
9-gram	0.8906	0.8889	0.8875		9-gram	0.4794	0.498	0.4922	
*Q*-gram	0.9368	0.9441	0.9500		*Q*-gram	0.7698	0.7953	0.7645	
Multiple	0.9472	0.9591	0.9621		Multiple	0.8091	0.8225	0.7892	

Note that Linkage kernel considers the linkage similarity among *q*-grams, KCaM kernel considers the structure similarity among *q*-grams, while linkage KCaM kernel considers both. The left of Table 4 reports the results of the classification for leukemic cells. The result shows that all the three kernels for weighted *q*-gram methods preforms better than traditional *q*-gram. In particular, for smaller values of *q*, the weighted kernels obtain higher accuracy, supporting the fact that it has been shown that trimer (tri-saccharide) structures have been most effective in discriminating between glycans related to leukemic cells compared to non-leukemic blood components. The right of Table 4 reports the results of cystic fibrosis. The AUCs of all the methods indicate that KM kernel performs best for all the methods. This implies that *q*-gram structure similarity contributes to cystic fibrosis-related glycan classification. In contrast to the leukemic cell data set, the result that linkage and Linkage KCam method performs worse than traditional *q*-gram method for lower values of *q *indicates that the *q*-gram similarity obtained from comparing their glycosidic linkages may not be helpful for the classification of cystic fibrosis. This result is supported by the fact that it has been shown in previous work that the sulfate-GlcNAc dimeric structure, which does not contain any glycosidic bond data, is a potential glycan biomarker. Interestingly, the best performance of KCaM kernel especially for higher values of *q *indicate that larger structures may be overlooked as potential biomarkers, thus illustrating the effectiveness of weighted *q*-gram method in this classification task. In conclusion, the results for two glycan data sets both show the improved prediction accuracy of the weighted *q*-gram method for glycan biomarker prediction.

## Discussion

In this paper, we focused on glycan classification by considering the similarity among *q*-gram. In the traditional *q*-gram method, similarity among sub-structures of glycans (*q*-gram) is not considered. Thus it assumes that *q*-gram similarity contributes nothing in glycan classification. We proposed a weighted *q*-gram method based on three kernels including linkage kernel, KCaM kernel and linkage KCaM kernel, and then compared the performance of weighted *q*-gram method with that of the traditional *q*-gram method using two glycan data sets. The results show that the consideration of similarity among *q*-grams contributes to higher accuracy of classification. Further research may focus on the biological properties of the glycan structure features that contribute to the increase in performance of these kernels, thus aiding the understanding of the mechanisms behind glycan structure recognition.

## Conclusion

We proposed a new approach in this paper to classify glycans based on their tree structures. The method attempts to involve similarity among *q*-grams in glycan classification. Three kernels (Linkage, KCaM and Linkage KCaM) are constructed for weighted *q*-gram methods. The experimental results showed its effective role in classification of glycan functions.

## Competing interests

The authors declare that they have no competing interests.

## Authors' contributions

LL and KFA conceived the idea and designed the research. TY processed the data. LL performed the research and analyzed the results. LL, KFA and WC wrote the paper. All authors read and approved the final manuscript.
